# A Multi-Scale Target Detection Method Using an Improved Faster Region Convolutional Neural Network Based on Enhanced Backbone and Optimized Mechanisms

**DOI:** 10.3390/jimaging10080197

**Published:** 2024-08-13

**Authors:** Qianyong Chen, Mengshan Li, Zhenghui Lai, Jihong Zhu, Lixin Guan

**Affiliations:** College of Physics and Electronic Information, Gannan Normal University, Ganzhou 341000, China; qychen@gnnu.edu.cn (Q.C.); 1220814006@gnnu.edu.cn (Z.L.); zhujihong@gnnu.edu.cn (J.Z.); lxguan@gnnu.edu.cn (L.G.)

**Keywords:** DIoU, improved Faster R-CNN, multi-scale target detection, ResNet101, Soft-NMS

## Abstract

Currently, existing deep learning methods exhibit many limitations in multi-target detection, such as low accuracy and high rates of false detection and missed detections. This paper proposes an improved Faster R-CNN algorithm, aiming to enhance the algorithm’s capability in detecting multi-scale targets. This algorithm has three improvements based on Faster R-CNN. Firstly, the new algorithm uses the ResNet101 network for feature extraction of the detection image, which achieves stronger feature extraction capabilities. Secondly, the new algorithm integrates Online Hard Example Mining (OHEM), Soft non-maximum suppression (Soft-NMS), and Distance Intersection Over Union (DIOU) modules, which improves the positive and negative sample imbalance and the problem of small targets being easily missed during model training. Finally, the Region Proposal Network (RPN) is simplified to achieve a faster detection speed and a lower miss rate. The multi-scale training (MST) strategy is also used to train the improved Faster R-CNN to achieve a balance between detection accuracy and efficiency. Compared to the other detection models, the improved Faster R-CNN demonstrates significant advantages in terms of mAP@0.5, F1-score, and Log average miss rate (LAMR). The model proposed in this paper provides valuable insights and inspiration for many fields, such as smart agriculture, medical diagnosis, and face recognition.

## 1. Introduction

Object detection has long been a research focus for computer vision, and it has been extensively applied in areas such as face recognition, medical image diagnosis, and road detection [1,2,3]. At present, deep learning-based target detection methods can be broadly categorized into two main classes [4,5]. One class is the two-stage object detection approach typified by Region-based Convolutional Neural Networks (R-CNNs), and the other class is the one-stage approach typified by You Only Look Once (YOLO). These two types of algorithms have their own characteristics and advantages [6]. One-stage target detection algorithms detect targets directly on the original image without a region proposal step, so these algorithms are relatively faster, but the detection accuracy decreases when detecting different multi-scale targets. Two-stage object detection algorithms exhibit relatively higher detection accuracy but at the cost of slower processing speeds. Driven by the rapid advancement of deep learning, target detection algorithms have achieved impressive gains in both accuracy and processing speed. R-CNN [7] is the seminal work in object detection algorithms, which computes over candidate regions generated by the selective search method, and further applies SVM classification and bounding box regression. Consequently, R-CNN consumes too much time in image processing, resulting in low detection efficiency. He et al. [8] introduced the Faster R-CNN on the basis of R-CNN, which introduced the Region Proposal Network (RPN) to generate candidate regions and utilized shared convolutional features to further improve detection accuracy and efficiency. Cai et al. [9] introduced Cascade R-CNN, which is best characterized by cascading classifiers and multi-stage training to improve detection accuracy and speed. Wan et al. [10] proposed an improved version of Faster R-CNN with optimized convolutional and pooling layers for detecting a wide range of fruits and achieving higher accuracy. Yang et al. [11] introduced an improved strawberry detection algorithm based on Mask R-CNN, which resulted in a substantial improvement in model generalization and robustness. After the R-CNN family of algorithms, Divvala et al. [12] proposed YOLO as an alternative to R-CNN. Unlike R-CNN, YOLO directly predicts classifications and regressions from features, using a single fully connected layer for both tasks. This design enhances speed and efficiency in processing. However, the disadvantage of YOLO is that its generalization ability and robustness are not strong, and it is easy to miss small targets. With the aim of improving the above problems effectively, Liu et al. [13] introduced the Single Shot MultiBox Detector (SSD) family of algorithms. Zhu et al. [14] used SSD to detect fruits on mango trees with an F1 of 0.91. Anagnostis et al. [15] used SSD to categorize infected trees in walnut orchards and the method detected whether walnut leaves were infected with 87% accuracy. Tian et al. [16] proposed EasyRP-R-CNN, a convolution-based framework for cyclone detection. The method was improved based on Region of Interest and achieved satisfactory detection accuracy. Li et al. [17] proposed a lightweight convolutional neural network, WearNet, to achieve automatic detection of scratches on contact sliding parts such as metal molding, and the classification accuracy of the method can reach 94.16%.

All of the methods mentioned above have some problems. (1) These methods detect and recognize a single or no target categories, which cannot satisfy the multi-target detection task, and are not able to accurately localize and recognize small targets. (2) These methods do not have strong feature extraction capabilities for small targets and cannot extract enough information about the target features. Thus, they can generate noise in the detection region, resulting in a decrease in accuracy. (3) These methods do not achieve a balance between detection accuracy and speed to meet the real-time demands of detection tasks. For the purpose of improving the detection accuracy in a multi-scale target environment, after considering accuracy and detection efficiency, this paper chooses to use Faster R-CNN as a baseline to detect different multi-scale targets on the Pascal VOC (Visual Object Classes) dataset [18]. In this paper, the following modifications are made while reducing model computation and improving model detection performance:

(1) ResNet101 [19] is employed as the trunk network in the improved Faster R-CNN, which enhances the feature extraction capabilities of the model.
(2) The Online Hard Example Mining (OHEM) algorithm [20] is used to help the model learn hard-to-classify samples more efficiently, which in turn enhances the model’s capacity for generalization. The Soft non-maximum suppression (Soft-NMS) algorithm [21] and the Distance Intersection Over Union (DIOU) algorithm [22] are used to optimize the excessive bounding boxes generated by the RPN and their overlap degree, which enhances the accuracy of detecting small targets and improves the issue of missed target detection.
(3) The RPN structure is optimized by adding an anchor box with a scale of 64 and using a smaller convolutional kernel to achieve bounding box regression. Employing the multi-scale training (MST) method to train the improved Faster R-CNN [23] achieves a balance between detection accuracy and speed.

## 2. Methodology and Modeling

In this chapter, we first introduce the overall structure of the proposed model. Then, we provide a detailed explanation of the improvements and integrated modules within the model. Finally, we briefly describe the performance evaluation metrics used in the experiments.

### 2.1. The Adjusted Faster R-CNN Network

**Figure 1:**
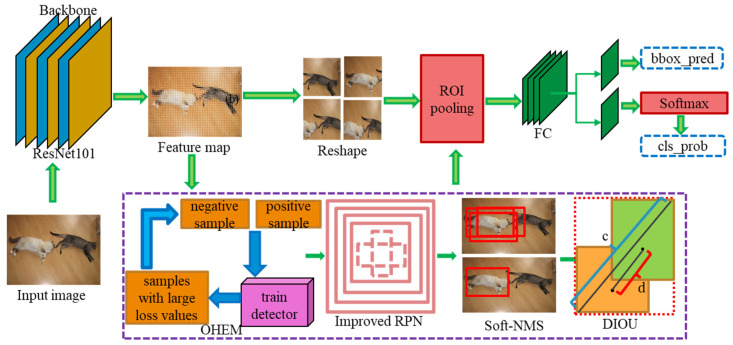
General framework of the improved Faster R-CNN.

Figure 1 shows the overall framework of the improved Faster R-CNN [24]. By comparing the four networks, VGG16, ResNet34, ResNet50, and ResNet101 [19], ResNet101 is chosen as the trunk network. The introduction of DIOU can increase the effectiveness of the improved Faster R-CNN regarding the problems of slow convergence of the target detection loss function and target regression localization accuracy. The introduction of OHEM enables the improved Faster R-CNN to mine difficult samples in the dynamic training process. This can improve the issue of imbalance between positive and negative samples during the training process. As depicted in Figure 1, the feature map from the ResNet101 trunk network is input into the optimized RPN. At this point, a large number of candidate proposal boxes are generated on the feature map. Soft-NMS is used to eliminate redundant target proposal boxes. It can reduce the miss rate of small targets by gradually decreasing the confidence score of overlapping proposal boxes.

#### 2.1.1. Improved Backbone Network

**Figure 2:**
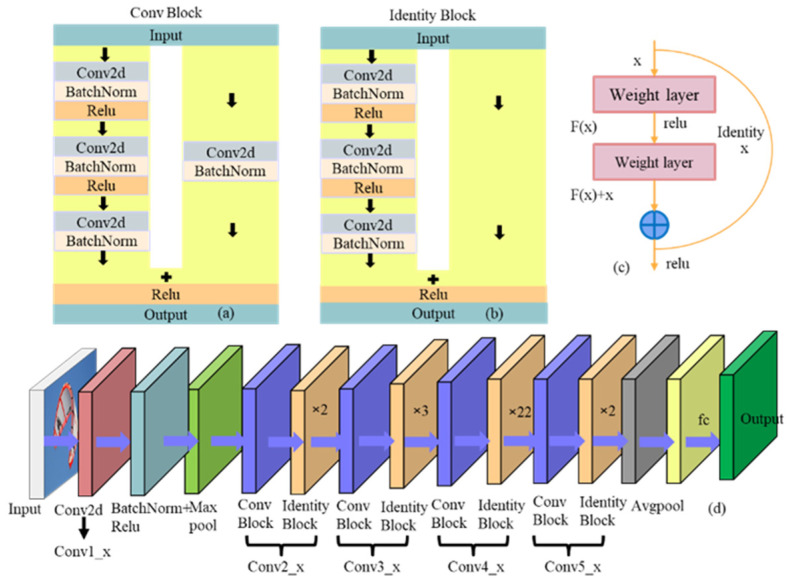
Resnet101 network composition. (**a**) Conv Block. (**b**) Identity Block. (**c**) Residual block. (**d**) Resnet101 network structure.

VGG16 [25] serves as the trunk network for the original Faster R-CNN. In general, data expansion and increasing network depth methods can be used to improve model performance, and network depth is very important for optimizing network performance [26]. ResNet [27] makes it possible for information to skip certain layers directly by introducing direct connections across layers in the network. This effectively improves the problems of gradient vanishing and gradient explosion. ResNet mainly uses convolutional operations instead of fully connected layers, which reduces the number of network parameters and can effectively avoid overfitting problems. Common ResNet structures are ResNet34, ResNet50, and ResNet101 [28]. One of the most significant features of ResNet50 compared to ResNet34 is the introduction of a new bottleneck residual block structure. It comprises a sequence of 1 × 1 convolutional layers, followed by a 3 × 3 convolutional layer, and then another 1 × 1 convolutional layer. This structure allows ResNet50 to have stronger feature representation while maintaining the depth of the model. ResNet101 has an additional set of convolutional blocks compared to ResNet50, which contains multiple residual units. The deeper network structure allows ResNet101 to further enhance its expressive and learning capabilities, allowing it to better capture image details and semantic information, as shown in Figure 2.

ResNet101 consists of two fundamental blocks, as depicted in Figure 2a and Figure 2b, respectively, named Conv Block and Identity Block. The Conv Block’s input and output dimensions are different and cannot be connected in series. Its function is to change the network dimension. The input dimensions and output dimensions of the Identity Block are the same and can be connected in series to deepen the network. ResNet101 consists of multiple residual units in each convolutional block, as illustrated in Figure 2c. Each residual unit performs three convolutional operations. The shortcut connections of residual units and the identity mapping help address the issues of gradient vanishing or exploding, which can lead to a decrease in detection accuracy. As shown in Figure 2d, ResNet101 consists of five convolutional layers and has a depth of 101 layers, which can provide stronger feature extraction capabilities.

In the fourth chapter, comparative experiments are conducted for VGG16, ResNet34, ResNet50, and ResNet101. Experimental results indicate that ResNet101 outperforms the other three networks in overall detection performance. Therefore, ResNet101 has been selected as the trunk network for the improved Faster R-CNN.

#### 2.1.2. Modifying the Region Proposal Network

**Figure 3:**
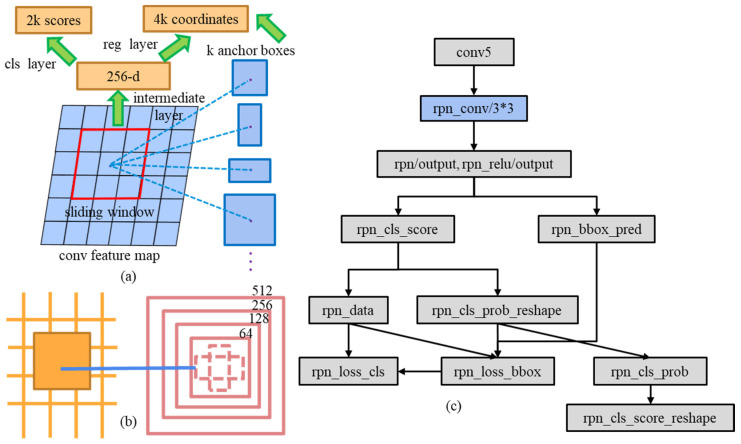
Optimized RPN schematic. (**a**) RPN sliding window and anchors. (**b**) Modified RPN anchors. (**c**) Modified RPN.

In the object detection process, CNNs are usually used to extract image features. These features are convolved and pooled through several convolution and pooling operations to produce a smaller feature map (also referred to as an activation map). The activation map contains semantic information and location information taken from the image, and then the activation map is input into the RPN. The RPN [8,29] will first further extract features from the input activation map through a shared convolutional layer, and then it will initially generate a set of predefined anchor boxes at each spatial position of the activation map to accomplish object detection. These predefined anchor boxes generated during the detection process usually have different width-to-height ratios as well as areas. Figure 3 shows the schematic of the optimized RPN.

For each anchor box, the RPN network uses a binary classifier to predict whether it contains a target. It outputs the probability that each anchor box belongs to the foreground or background to complete the initial classification prediction. The conventional RPN generates nine anchor boxes at each spatial position of the activation map with aspect ratios of 1:1, 1:2, and 2:1 and scales of 128, 256, and 512, respectively, as shown in Figure 3a. In order to improve the accuracy of detecting small targets and reduce the leakage rate, a new anchor box with a scale of 64 is added to the RPN while the aspect ratio remains unchanged [30], so that there are a total of 12 anchor boxes at each position of the feature map, which is then able to better capture small-scale targets, as shown in Figure 3b. In addition to the classification predictions, the RPN completes an initial bounding box regression on the positive samples (containing the target’s anchor boxes) with the goal of accurately predicting the target’s bounding box position. The output is the translation and scaling parameters relative to each positive sample anchor box. As depicted in Figure 3c, the structure of the RPN is simplified, and only the 3 × 3 convolutional kernel is used to generate 256 feature maps. The benefits of using a 3 × 3 convolution kernel include: reducing the number of parameters and computational burden, improving computational efficiency, capturing local features, effectively utilizing boundary information, and simplifying the network design. The 3 × 3 convolution kernel is able to maintain model simplicity and computational efficiency while ensuring the effectiveness and accuracy of feature extraction.

#### 2.1.3. Optimization of Detection Boxes and Sample Imbalance

In object detection, the bounding box mechanism is used to locate and identify objects in an image using rectangular boxes. This is mainly achieved by predicting the center coordinates, width, and height of the bounding boxes to ensure accurate object localization and classification. Regression loss functions and post-processing algorithms are employed to optimize the position of the bounding boxes and remove redundant detections, enhancing the accuracy and efficiency of detection. In the improved Faster R-CNN, several common strategies are adopted to enhance the bounding box mechanism. Below is a brief introduction to these strategies.

(1) DIOU

**Figure 4:**
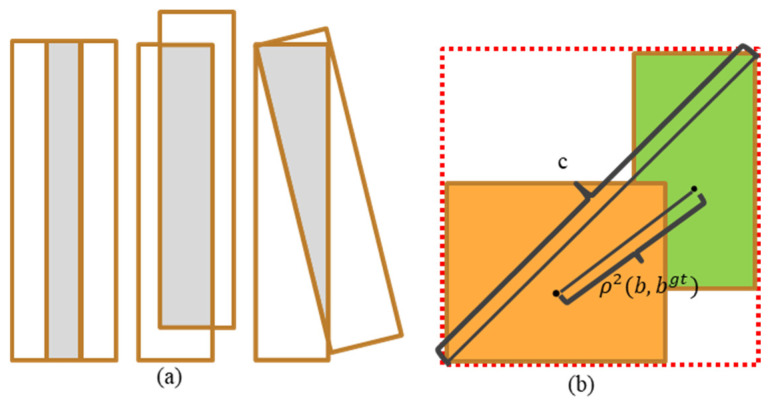
Schematic representation of the different levels of overlap of the bounding box. (**a**) Three possible scenarios when IoUs are identical. (**b**) DIOU loss for bounding box regression.

IOU [31] is a widely used performance index in object detection, indicating the degree of overlap between the predicted box and the actual box. Figure 4 shows the schematic diagram of the bounding box with different degrees of overlap. Currently, there are two main disadvantages to using IOU as a loss function for object detection. One is that if there is no overlap between the two detection boxes, IOU = 0, which means that the two detection boxes can no longer participate in deep learning training. Secondly, it can be possible for two detection boxes to have different degrees of overlap but yield the same IOU. This means that IOU does not give accurate feedback on the degree of overlap between the detection boxes, as shown in Figure 4a.

With the aim of improving the above two problems effectively, DIOU [22] is used to better refine the positioning of the bounding box. DIOU can effectively address the issues of slow convergence and regression localization accuracy that exist in IOU, and its formula is defined as Equation (1).

(1) $$L_{DIOU}=1-DIOU=1-(IOU-\frac{\rho^{2}(b,b^{gt})}{c^{2}})=1-IOU+\frac{\rho^{2}(b,b^{gt})}{c^{2}}$$

where $\rho^{2}(b,b^{gt})$ denotes the distance between the centers of the prediction box b and the ground truth $b^{gt}$, and c denotes the diagonal length of the smallest region that contains both the prediction box and the ground truth, as shown in Figure 4b. Since the DIOU loss function has the characteristic of being able to minimize the distance between two prediction boxes directly, it converges and regresses faster than IOU. DIoU improves the traditional IoU [32] metric by addressing the insensitivity of IoU to the distance between bounding boxes. By introducing a penalty term in the loss function based on the center distance of the bounding box, DIoU provides a more comprehensive and accurate measure of bounding box similarity, which leads to better performance in target detection tasks. Using DIOU as the loss function of the improved Faster R-CNN can make the model pay more attention to hard-to-detect targets during the training process, which is especially important for the detection of small and irregularly shaped targets.

(2) OHEM

During the object detection training process, the activation maps from the trunk network are input into the RPN, at which time a large number of candidate proposal boxes are generated. Generally, the IOU threshold is set to 0.5 and proposal boxes with an IOU above 0.5 are retained as positive samples, while those below 0.5 are treated as negative samples. This leads to significantly more positive samples than negative ones. As a result, the model may overlook difficult negative samples that are challenging to detect. These difficult samples can contribute higher loss values, improving the overall detection performance of the model.

**Figure 5:**
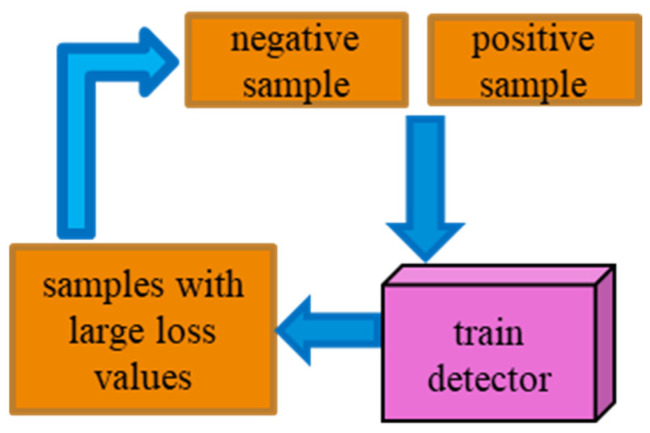
Diagram of the OHEM algorithm.

In order to enable more difficult samples to be used in the dynamic process of training, OHEM is introduced into the improved Faster R-CNN [20]. Figure 5 illustrates the working principle of OHEM. OHEM [33] improves the training effect of the model by dynamically selecting and processing those difficult samples that the model currently has difficulty classifying during the training process. Specifically, it first detects the input image during each training session and selects the samples with higher loss as difficult samples according to the loss value. These difficult samples are then utilized for backpropagation and parameter updating so that the model learns to correctly classify difficult cases faster, thus improving its overall performance. The OHEM training strategy in the dynamic process of training can send the target samples that are difficult to detect into the network again for deep learning training. This makes the network more sensitive to the detection target, which in turn improves the target detection accuracy.

(3) Soft-NMS

NMS is a very important algorithm in target detection. Its basic idea is to sort the proposal boxes by their confidence scores and retain the one with the highest score. In this process, if the overlap between two proposal boxes exceeds a set threshold (generally 0.5), the box with the lower score is discarded, and the one with the higher score is retained. Therefore, The NMS score is based solely on the classification confidence, without considering the localization accuracy of the bounding box. This means that the classification and localization confidences are not positively correlated.

To effectively address some of the limitations of NMS, Soft-NMS is adopted in the improved Faster R-CNN [21]. Its linear weighted equation is defined as Equation (2).

(2) $$S_{i}=\left\{\begin{array}{cc} S_{i} & \;IOU(M,b_{i})<N_{t}\\ S_{i}(1-IOU(M,b_{i}) & \;IOU(M,b_{i})\geq N_{t} \end{array}\right.$$

Here, $S_{i}$ is the proposal box confidence score and M is the proposal box with the highest score. The set of all proposal boxes during training is denoted by b, the area of overlap between M and the proposal boxes in set b is denoted by $IOU(M,b_{i})$, and $N_{t}$ is the IOU threshold set at the beginning. The difference between Soft-NMS and NMS is the way overlapping bounding boxes are handled. Traditional NMS directly removes other bounding boxes that overlap with the highest scoring box above a certain threshold, which may result in some correct detections being mistakenly removed. In contrast, Soft-NMS does not remove these overlapping bounding boxes, but gradually reduces their scores. Soft-NMS works by attenuating the scores proportionally to the degree of overlap between the bounding box and the highest-scoring box [34]. The larger the overlap, the more the score is attenuated. In this way, Soft-NMS is able to retain more valuable detection results, reduce missed detections, and improve the accuracy of target detection.

#### 2.1.4. Multi-Scale Training

**Figure 6:**
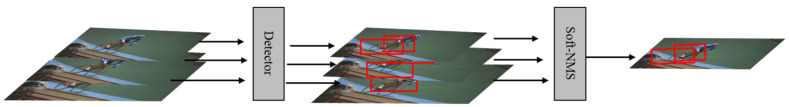
Diagram of the multi-scale training strategy.

The MST [23] method can enhance the model’s adaptability and generalization capabilities when detecting a variety of target sizes. An image pyramid called MST [35] is used in the training process of CNN. As shown in Figure 6, image training is achieved by randomly inputting images of different scales within a given segment of image size, which enables the target detection model to adapt to targets of different scales. In the testing phase, the same image at different scales is input for multiple detections. Finally, Soft-NMS is employed to integrate all detection results, which enables the detection model to cover as many targets as possible at multiple scales and improves the robustness of the detection model. During the feature extraction phase, the generated activation map will be significantly smaller than the original image. This can make it challenging for the model to focus on the details of small targets. Therefore, by providing the model with larger and richer images, its detection capabilities can be enhanced effectively [36]. In this paper, MST is used to train the improved Faster R-CNN. The training samples have image lengths ranging from 380 to 640, and image widths spanning 300 to 450.

### 2.2. Evaluation Metrics

To assess the model’s detection capabilities, several widely used evaluation metrics are used here [37,38]. These metrics include Average Precision (AP), F1 Score (F1), Log average miss rate (LAMR), and mean Average Precision (mAP). The specific formula is given in Equation (3).

$X_{TP}$ represents the number of targets accurately identified by the model, $X_{FP}$ indicates the number of targets mistakenly identified by the model, and $X_{FN}$ denotes the number of targets missed by the model. AP is the area surrounded by the PR curve. LAMR is used to measure the miss rate of the target. mAP is used as a comprehensive evaluation metric, and its value is the average of the APs of all detection categories.

(3) $$\left\{\begin{array}{c} precision(P)=\frac{X_{TP}}{X_{TP}+X_{FP}}\times 100\%\\ recall(R)=\frac{X_{TP}}{X_{TP}+X_{FN}}\times 100\%\\ AP=\int_{0}^{1}p(r)dr\\ F1=\frac{2PR}{P+R}\times 100\%\\ LAMR=\left(\frac{1}{M}\sum_{i=1}^{M}\log(MR_{i})\right)\\ mAP=\frac{\sum_{i=1}^{K}AP_{i}}{K}\times 100\% \end{array}\right.$$

## 3. Experiments and Results

In this chapter, we first introduce the datasets used in the experiments. Then, we provide a detailed explanation of the data augmentation methods applied to these datasets. Next, we conduct experiments on datasets with different proportional distributions and present the recorded results.

### 3.1. Data and Preprocessing

#### 3.1.1. Data Collection

To accentuate the model’s performance more prominently, this experiment uses the Pascal VOC [18] dataset, which contains a total of 20 detection categories. Each target has three types: large, medium, and small, which can fulfill the purpose of detecting different multi-scale targets, as illustrated in Table 1. The Pascal VOC 2007 dataset provides rich object classes and diverse scenes, which makes it an important benchmark for evaluating target detection and other computer vision tasks [39]. It covers a wide range of categories from humans and animals to vehicles and indoor objects, with diverse lighting conditions, viewing angles, and occlusions, which makes it an important tool for testing the robustness and generalizability of algorithms. The numbers 1–20 for subsequent related experiments correspond to the detection targets in the table.

#### 3.1.2. Data Augmentation

**Figure 7:**
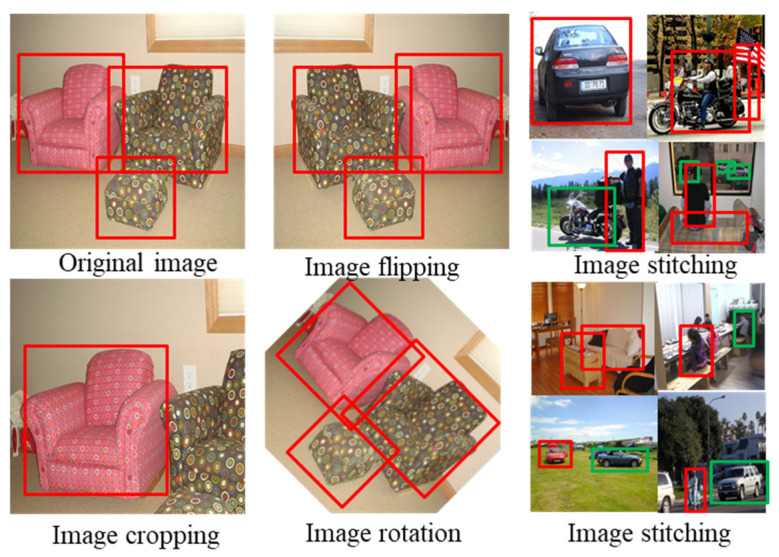
Data enhancement effect diagram.

Before training the model, after analyzing the dataset, it was found that the dataset was very uneven and that the image size was not uniform. Therefore, the dataset needed to be processed in the preprocessing stage. Image augmentation is a widely adopted technique to expand the dataset and strengthen the model’s robustness [40,41]. Data distribution can be changed regularly by image enhancement techniques, weakening the features of unimportant objects. There are two types of commonly used image enhancement methods. One is image enhancement based on image processing techniques and the other is deep learning based image enhancement algorithms. This paper adopts the first one, which mainly includes rotation, flipping, random cropping, and mosaic stitching. In this case, mosaic stitching is the proportional recombination of four images from the training set into a new image. This enhances the object’s contextual background, allowing the model to learn richer features from a single image. The effect of data enhancement is shown in Figure 7. After data enhancement, the dataset was expanded from 9963 to 15,000 images. The image size is uniformly resized to 400 × 400.

### 3.2. Results

All experiments are conducted on the Colab cloud server platform, using a Tesla T4 graphics card with 16 GB of video memory and Windows as the operating system. The deep learning framework used is PyTorch 1.9, with 100 training epochs and all images uniformly resized to 400 × 400. To improve model robustness and efficiency, the expanded dataset is divided into three categories, A, B, and C [30], and the proportional allocation of the dataset in each category is depicted in Table 2.

**Figure 8:**
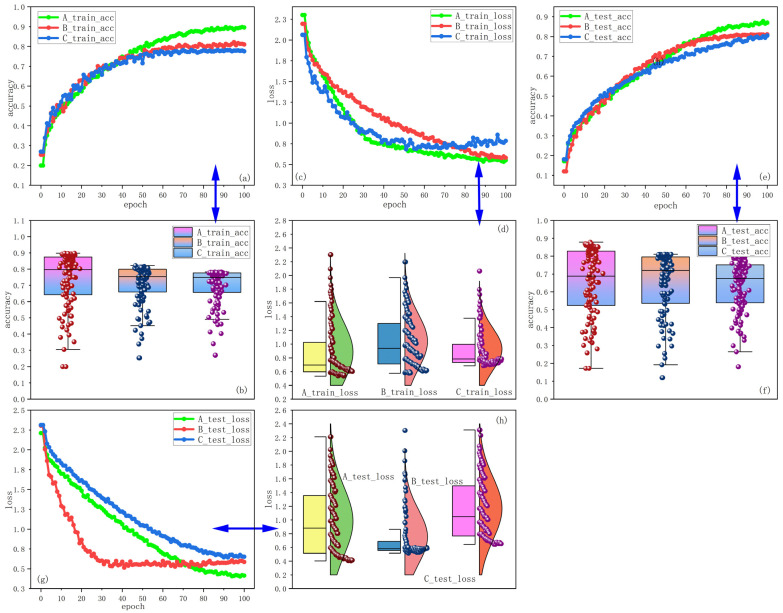
The result curves and data distribution for datasets A, B, and C. (**a**) Training accuracy curve. (**b**) Distribution of training accuracy data. (**c**) Training loss curve. (**d**) Distribution of training loss data. (**e**) Test accuracy curve. (**f**) Distribution of test accuracy data. (**g**) Test loss curve. (**h**) Distribution of test loss data.

The improved Faster R-CNN is first applied to the training set of three datasets and the results are recorded. During the training process, model error is minimized by adjusting various training parameters over 100 iterations of training. At last, the improved Faster R-CNN is trained using test data from the three datasets. Figure 8 shows the experimental result curves and data distributions during the training process.

By observing the changes and positions of the curves, we know that the accuracy curves of dataset A are steadily increasing and located at the top of the three curves. This indicates that the improved Faster R-CNN has the best performance on dataset A. Meanwhile, the loss value curve of dataset A decreases steadily. The fluctuation is small, located at the bottom of the three curves, and gradually tends to be stable. This also indicates that the improved Faster R-CNN has stronger stability and robustness on dataset A. The variation of these curves can be seen in Figure 8a,c,e,g. Accordingly, by observing the data distribution of the experimental results, it can be found that, compared to the training and testing sets of datasets B and C, the accuracy values of dataset A are more centrally distributed and have higher positions, while the loss values are at lower distribution positions and more compactly distributed. The improved Faster R-CNN is adequately trained on dataset A with better generalization performance. This is because 80% of the images in dataset A are used for training so that the model can extract richer information about image features. Based on these experimental results, dataset A is used for all subsequent experiments.

## 4. Discussion

### 4.1. Comparison of Trunk Networks

In this section, comparative experiments [24] are conducted for VGG16, ResNet34, ResNet50, and ResNet101. A total of 15,000 images of dataset A are divided in the ratio of 8:1:1, and SGD is employed to optimize the model [42]. The momentum is 0.9, the learning rate is 0.001, and the F1 threshold is 0.5. After 100 epochs, the model training curve converges, indicating that the model has reached an optimal solution. The improved Faster R-CNN improves the capability of target detection by combining different trunk networks. Table 3 shows the experimental results on the test set.

As can be seen in Table 3, the trunk network with a residual structure has stable detection performance in the multi-scale target category. With the increase of network layers, the detection accuracy of ResNet50 surpasses that of VGG16 and there are fewer missed detections for targets. Since the residual unit module inside the ResNet101 network is connected by a multilevel residual network, this makes it better able to capture the local feature information of multi-scale targets. ResNet101 has the best detection effect, with mAP@0.5 reaching 74.9% and the LAMR reaching 29.5%. Therefore, from Table 3, it can be seen that the improvement of the Faster R-CNN trunk network is effective. ResNet101 performs better in multi-scale target category detection, and the miss rate for small targets is lower.

**Figure 9:**
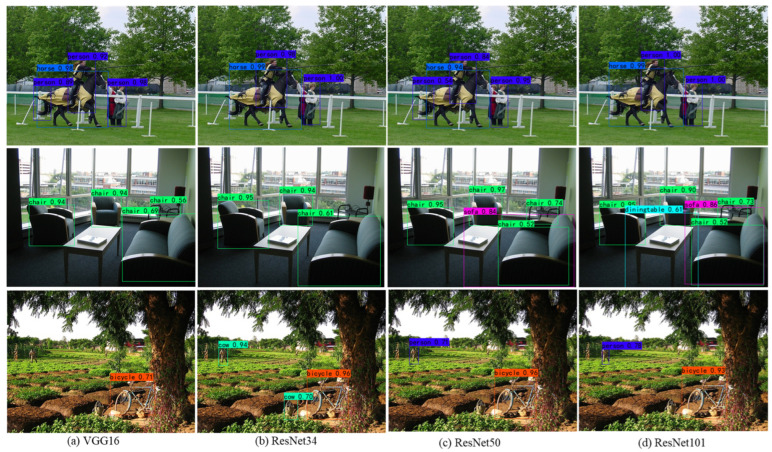
Visual comparison of detection results for four backbone networks.

Figure 9 compares the detection results of the four trunk networks. Focusing on the first two columns, when using ResNet101 as the feature extractor, detection performance is notably improved compared to the other trunk networks. The target detection scores are generally higher, the target localization is more accurate, and there are no target misdetections or missed detections. VGG16 and ResNet50 mistakenly detected the ponytail as a person, while ResNet34 missed the couch and did not detect the chair in the upper right corner. In the third resultant image containing only small targets, ResNet101 can accurately detect the person and the occluded bicycle in the image. This further demonstrates better detection performance when using ResNet101.

### 4.2. Comparison of Different Object Detectors 

**Figure 10:**
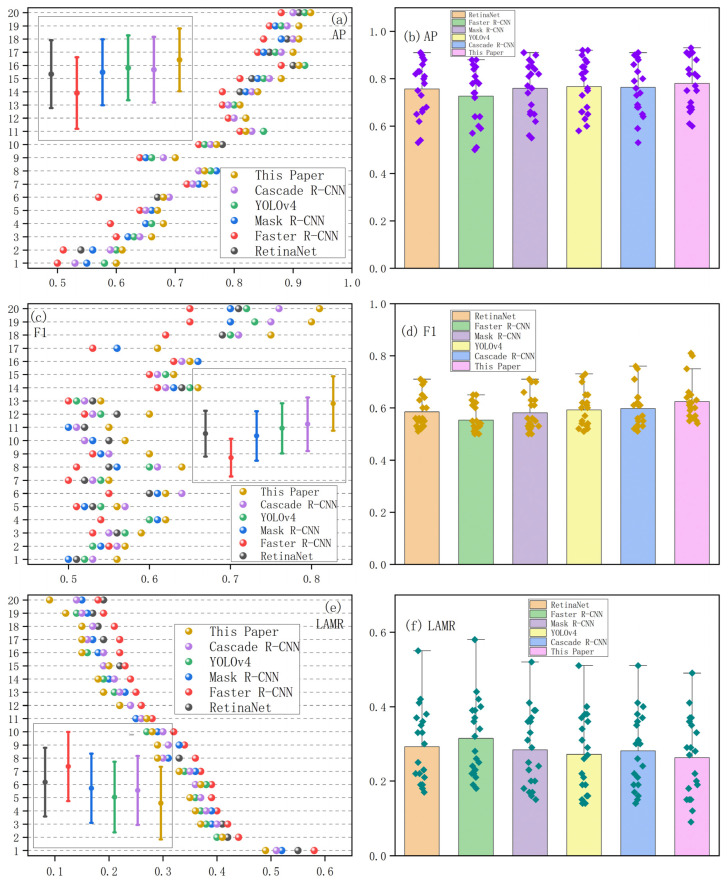
Comparison of experimental result values and their data distribution for 20 detection targets for each comparison model. (**a**,**b**) Comparisons of AP values. (**c**,**d**) Comparisons of F1 values. (**e**,**f**) Comparisons of LAMR values.

To demonstrate that the proposed method has better detection effectiveness and accuracy, experiments are also performed on the test set of the expanded dataset for RetinaNet [43], Faster R-CNN [8], Mask R-CNN [44], YOLOv4 [45], and Cascade R-CNN [9]. The SGD optimizer is used to optimize the model with a momentum of 0.9 and a learning rate of 0.001, with the learning rate decaying 0.1 times every 20 epochs. By continuously adjusting the training parameters, after 100 epochs, the training curves of each comparison model gradually level off. This indicates that the model training process is relatively smooth. Table 4 shows the mean values of each metric for the 20 targets detected by the six comparison models on the test set. Figure 10 shows a comparison of the metric values and their data distributions for the 20 detection targets of the six comparison models on the test set.

Table 4 shows the mean values of each metric for the 20 targets detected by the six comparison models on the test set. Figure 10 shows a comparison of the metric values and their data distributions for the 20 detection targets of the six comparison models on the test set. The improved Faster R-CNN improves mAP@0.5 to 77.8%, F1 to 60.6%, and the LAMR to 26.5%. Compared to the other five detection models, the proposed method has better performance. Specifically, compared with the original Faster R-CNN, the mAP@0.5 is improved by 5.1%, the F1 is improved by 5.3%, and the LAMR is reduced by 4.9%. This indicates that the proposed method is effective in improving the detection rate of small targets as well as reducing the miss rate. In terms of detection speed, the proposed approach is 0.016 s slower than Faster R-CNN. The potential reason may be due to the increase in the number of anchor boxes in the RPN, which has resulted in a longer overall detection time for the proposed approach, slightly decreasing speed but obtaining more accurate detection results. This helps to achieve a balance between time and accuracy. 

**Figure 11:**
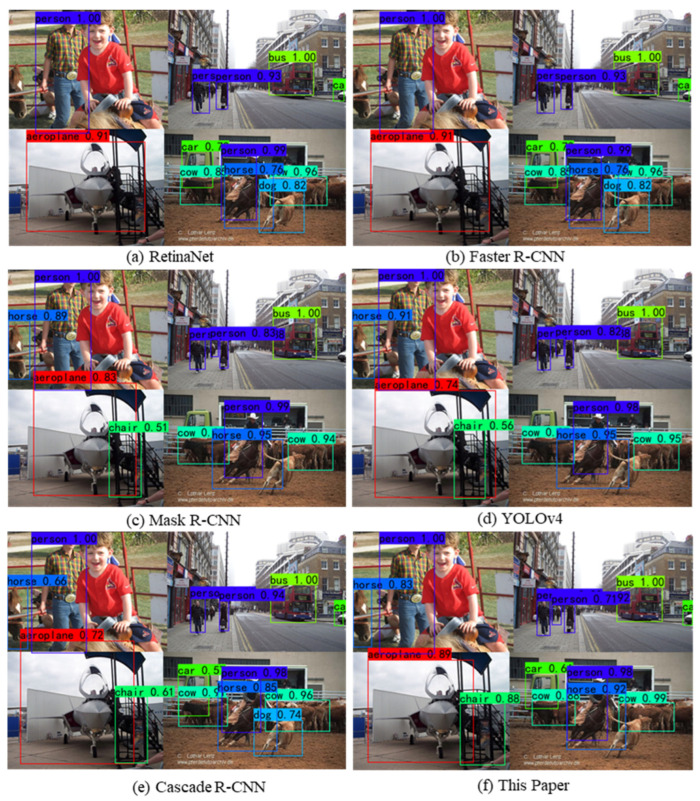
Visual comparison of detection results for six comparison models.

Observing Figure 10a,b, it can be seen that 15 AP values of the proposed approach are located in the first position, and the data distribution of the prediction results is more centralized than the other compared models. This indicates that the overall detection accuracy of the model is higher. From Figure 10c,d, it can be seen that 17 F1 values are in the leading position, among which two F1 values exceed 0.8, and the distribution intervals of the F1 values are relatively higher. This indicates that the model is more adaptable when facing multi-scale target categories. As shown in Figure 10e,f, the prediction results of the proposed approach all have LAMR values below 0.5, and the miss rate is also reduced for small targets such as pottedplant (1), sheep (4), and bottle (5). The reason for this may be that the bounding box optimization mechanism, as well as the introduced multi-scale training strategy, plays a role. The data distribution interval of the LAMR is also relatively lower. This indicates that the model is able to focus on small targets that are difficult to detect when performing multi-scale target category detection, and the model is more robust and stable. Figure 11 shows the visualization of the detection image after mosaic data enhancement. Compared to the other detection models, although the proposed approach is not the best in each target category, the overall effect is excellent. The very small car in the upper right corner, as well as the occluded chair in the lower left corner, can be accurately detected, and both target detection scores are relatively quite high. This demonstrates that the proposed approach can better adapt to the different sizes and shapes of targets in different multi-scale detection target environments and improve the accuracy of small target detection.

### 4.3. The Ablation Experiments and Analysis

In this section, we set up seven sets of ablation experiments on the test set to verify the validity of each module. The setting of the ablation experiments is detailed in Table 5. The SGD optimizer is used to optimize each combined model [42]. The momentum is 0.9 and the batch size is 64. To mitigate overfitting during training, the regularization parameter is 0.01 and the learning rate is 0.001. The number of training times for each combination of models is still 100 epochs, and to achieve timely adjustments to the training parameters, the training results are taken every 10 epochs. When the training curves of each combined model gradually level off, it indicates that the model can converge stably. The results of the ablation study are presented in Table 5.

**Figure 12:**
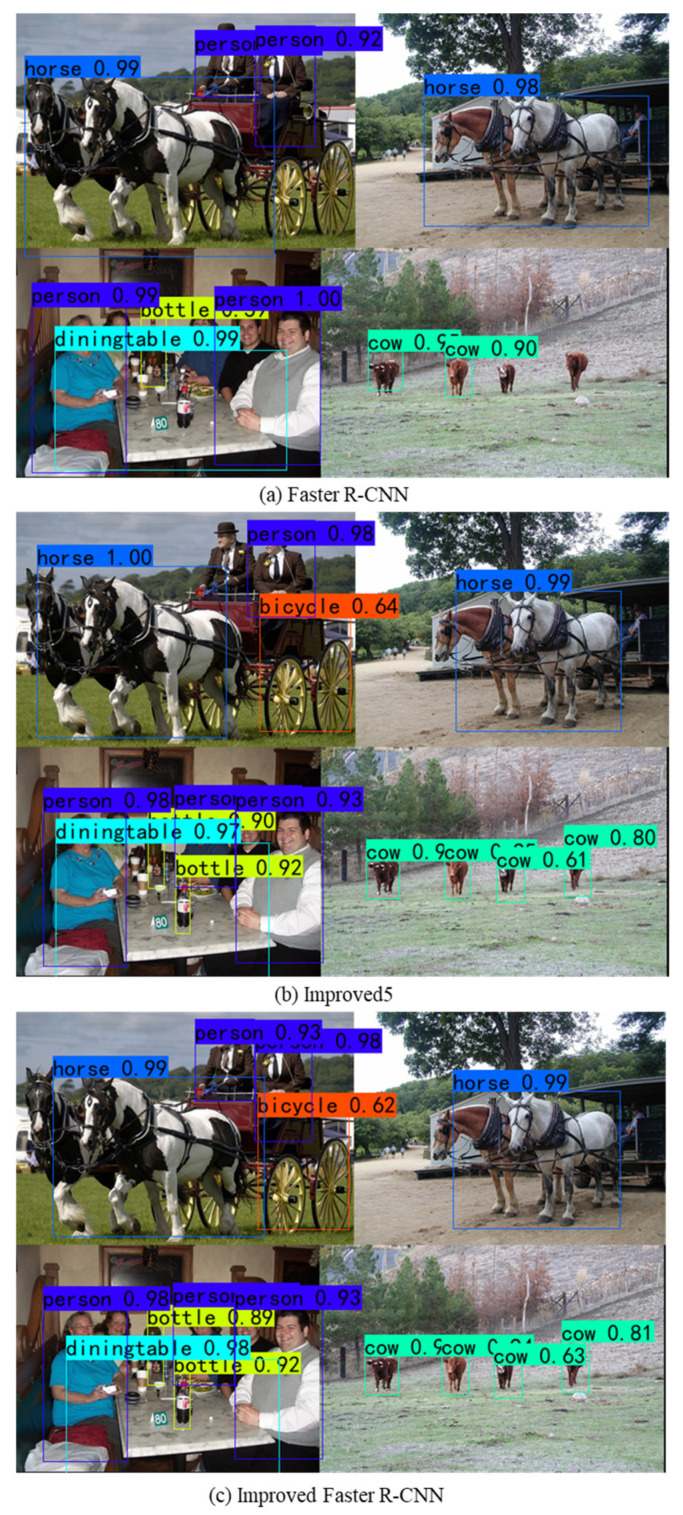
Visual comparison of detection results for ablation experiments.

As depicted in Table 5, the addition of the modified RPN to the Faster R-CNN (ResNet101) increases the mAP@0.5 by 0.3% and the F1 by 0.2%. The introduction of DIOU improves the mAP@0.5 by 0.2% and the F1 by 0.1%. It can be seen that the introduction of OHEM increases the mAP@0.5 by 0.5% and the F1 by 0.4%. This shows its improvement for the problems of sample imbalance and insufficient training of hard case samples in the target detection task. After replacing NMS with Soft-NMS, the miss rate is reduced, while performance and accuracy are improved. Finally, by combining the modules with a multi-scale training strategy, an improved Faster R-CNN is obtained with a mAP of 77.8%, which is 5.1% higher than the original Faster R-CNN, and the F1 is improved by 5.3%. It is worth noting that the LAMR of the proposed approach compared to Improve5 is lower. This improvement is attributed to the efficacy of the MST [16] in reducing the miss rate and enhancing the model’s recognition capabilities for small targets. The partial detection results for Faster R-CNN, Improved5, and Improved Faster R-CNN are shown in Figure 12. A comparison shows that Faster R-CNN is not very effective in detecting cows and bottles with small sizes and misses some small targets. While Improved5 improves this phenomenon, the network can detect some small targets that were originally missed. The best detection was achieved by Improved Faster R-CNN, which had more accurate edge localization, detected more small targets, and largely correctly identified overlapping and low-contrast targets. The detection performance of this model is much stronger.

## 5. Conclusions

This paper proposes a novel two-stage object detection model for detecting multi-scale objects from diverse categories. The model introduces improvements to the Faster R-CNN and its trunk feature extraction network. DIOU, OHEM, and Soft-NMS are used to improve the problems of unbalanced positive and negative samples and target miss rate during model training. The RPN is also optimized and the proposed approach is trained by employing a multi-scale training strategy. Comparison experiments with trunk networks verify that using the ResNet101 feature extraction network is more advantageous. The validity of the proposed approach is further confirmed by comparison experiments with other detection models. Ablation experiments are also conducted to verify that the modules in the proposed approach can indeed be useful. The experiments show that the proposed method has a mAP@0.5 of 77.8% and an F1 of 60.6%, which are 5.1% and 5.3% higher than the original Faster R-CNN, respectively. The experimental results show that the proposed method can improve the accuracy and performance of object detection in a multi-scale target detection environment. In the future, we will further optimize, extend, and experiment on more datasets so that the model can be better applied to different types of object detection scenarios and provide a roadmap for continued advancements in the field of multi-scale object detection.

## Figures and Tables

**Table 1:** Dataset distribution of Pascal VOC 2007.

Object Classes	Train	Validation	Test	Total
Pottedplant	133	112	224	469
Chair	224	221	417	862
Sofa	111	118	223	452
sheep	48	48	97	193
bottle	139	105	212	456
diningtable	97	103	190	390
Bird	180	150	282	612
boat	81	100	172	353
aeroplane	112	126	204	442
motorbike	120	125	222	467
tvmonitor	128	128	229	485
person	1025	983	2007	4015
train	127	134	259	520
bicycle	116	127	239	482
Cow	69	72	127	268
Dog	203	218	418	839
Cat	163	174	322	659
Bus	97	89	174	360
Car	376	337	721	1434
horse	139	148	274	561
total	2501	2510	4952	9963

**Table 2:** Proportional distribution of the three datasets.

Data Set	Train	Validation	Test
A (8:1:1)	12,000	1500	1500
B (7:1.5:1.5)	10,500	2250	2250
C (6:2:2)	9000	3000	3000

**Table 3:** Predictive performance of different trunk networks.

Methods	mAP@0.5 (%)	F1 (%)	LAMR (%)
VGG16	72.7	55.3	31.4
ResNet34	73.3	56.4	30.8
ResNet50	73.8	56.2	30.2
ResNet101	74.9	57.2	29.5

**Table 4:** Experimental mean results for six comparison models.

Methods	mAP@0.5 (%)	F1 (%)	LAMR (%)	T (s)
RetinaNet	75.6	58.5	29.2	0.155
Faster R-CNN	72.7	55.3	31.4	0.147
Mask R-CNN	75.8	58.2	28.5	0.153
YOLOv4	76.5	59.2	27.2	0.132
Cascade R-CNN	76.2	59.7	28.1	0.139
**This Paper**	**77.8**	**60.6**	**26.5**	**0.163**

**Table 5:** Setting of ablation experiments and experimental results.

Method	ResNet101	RPN	DIOU	OHEM	Soft-NMS	MST	mAP@0.5 (%)	F1 (%)	LAMR (%)
Faster R-CNN(VGG16)							72.7	55.3	31.4
Faster R-CNN(ResNet101)	✓						74.9	57.2	29.5
Improve1	✓	✓					75.2	57.4	29.2
Improve2	✓		✓				75.1	57.3	29.3
Improve3	✓			✓			75.4	57.6	28.9
Improve4	✓				✓		75.0	57.4	29.3
Improve5	✓	✓	✓	✓	✓		75.5	58.1	28.7
**This paper**	✓	✓	✓	✓	✓	✓	**77.8**	**60.6**	**26.5**

## Data Availability

The dataset is available free of charge at kaggle. (https://www.kaggle.com/datasets/qianyongchen/dataset, accessed on 10 August 2024).
